# Yam Protects Immunocompromised Mice from Influenza Infection via the Gut–SCFA–GPCR–Immune Axis

**DOI:** 10.3390/nu18111793

**Published:** 2026-06-02

**Authors:** Qingjun Li, Xinyan Qu, Menglin Li, Yingying Song, Qi Xu, Quanbo Wang, Hongjing Dong, Xiao Wang, Qian Liu

**Affiliations:** 1 School of Pharmaceutical Sciences, Shandong University of Traditional Chinese Medicine, Jinan 250355, China; liqjxxhh@126.com (Q.L.); 17861171650@163.com (M.L.); 2 Key Laboratory of Traditional Chinese Medicine Classical Theory, Ministry of Education, Shandong University of Traditional Chinese Medicine, Jinan 250355, China; 3 Shandong Analysis and Test Center, Qilu University of Technology (Shandong Academy of Sciences), Jinan 250353, China; songyingying@qlu.edu.cn (Y.S.); xuqi8270@sina.com (Q.X.); qbwang@qlu.edu.cn (Q.W.); donghongjing_2006@163.com (H.D.)

**Keywords:** yam, immunodeficiency, gut microbiota, short-chain fatty acid, GPCRs, influenza virus

## Abstract

**Background/Objectives**: Immunodeficiency can be induced by a variety of factors, such as aging, stress and poor nutrition, and leads to increased susceptibility to infection and disease. The current research was conducted to determine the immunoenhancing potential of yam and its underlying mechanism in a murine model of cyclophosphamide (CTX)-induced immunosuppression. **Methods**: The gut microbial community and generation of short-chain fatty acids (SCFAs) in response to yam were analyzed by 16S rRNA sequencing and GC-MS. The immune cells in the spleen were analyzed using flow cytometry. GPR41/GPR43/GPR109A triple-knockout mice were used to demonstrate the critical involvement of SCFAs in mediating the protective effect of yam, and RNA-sequencing technology was applied to investigate the potential mechanism by which yam orchestrated the observed metabolic, immune and reparative responses. **Results**: Yam alleviated the decline in spleen and thymus indices and modulated the frequency of B cells and CD4^+^ and CD8^+^ T cells and promoted the production of IgA, IgG and IgM. Yam increased the secretion of cytokines in the intestine and upregulated the levels of claudin and ZO-1. Yam also increased the content of SCFAs and induced beneficial modifications to the gut microbiota composition. The immune-enhancing activity of yam was confirmed, as evidenced by a notable decrease in viral load in immunosuppressed mice inoculated with influenza virus and its capacity to mitigate inflammatory response in pulmonary tissues. **Conclusions**: This study suggests that yam enhances immunity by synergistically regulating the gut–immune axis, supporting its development as a functional food intervention in managing immunodeficiency conditions.

## 1. Introduction

The immune system is essential for recognizing and eliminating harmful pathogens, maintaining homeostasis and especially protecting against infections. However, the immune system can be compromised by multiple factors, such as aging, stress and poor nutrition, leading to immune dysfunction or reduced immune capacity [1,2]. As one of the infections that readily arises in the context of a compromised immune system, influenza can cause severe or even life-threatening symptoms in high-risk populations. Pandemics, epidemics and sporadic influenza outbreaks are associated with high mortality rates. Host defense relies primarily on orchestrated innate and adaptive immunity, making proper immune function crucial for protection against influenza infection [3,4].

It is increasingly recognized that the gut microbiota plays a pivotal role in the mechanisms underlying immunodeficiency. These microbial communities communicate directly with the host’s intestinal epithelium, releasing metabolites into the blood, regulating systemic metabolism and exerting regulatory effects on distant lymphoid organs such as the spleen and thymus [5,6,7]. This evidence underscores the important role of the gut microbiome–metabolite–organ axis in maintaining systemic immune balance. Studies have revealed that dietary fiber is critical for supporting gut microbial diversity and metabolic profiles, as well as a positive role for short-chain fatty acids (SCFAs) of microbial origin in preserving intestinal barrier health and reducing inflammation [8,9]. Additionally, dietary polysaccharides have been recognized as natural prebiotics that modulate the gut microbiota and promote SCFA production, thereby contributing to intestinal barrier integrity and immune regulation [10,11]. SCFAs have been shown to be key players in regulating the gut microbiota and have important biological functions, including maintaining energy levels, modulating pH within the colon and inside cells, regulating cellular ion transport, and upregulating genes involved in cellular proliferation and differentiation [12,13]. These findings suggest that high-fiber or high-polysaccharide intake represents a promising therapeutic strategy for immune system modulation in patients with immune dysregulation. In general, diet serves as a non-invasive means to modulate host immunity by regulating the gut microbiota and its metabolic output.

Yam is a fiber-rich tuber that serves as both a food and a medicine globally, especially for populations in tropical and subtropical regions [14]. Yam has been shown to exhibit several important biological activities, including strengthening the stomach and spleen and nourishing the lungs and kidneys. According to recent research, the gut microbiota ferments yam, producing SCFAs and modifying the structure of the gut microbiome. These changes may underlie the health-promoting effects associated with yam consumption [15]. Emerging evidence also supports the diverse health benefits of yam’s bioactive ingredients, including anti-diabetic, antioxidant, anti-tumor, anti-inflammatory, anti-obesity and hypolipidemic effects [16]. Moreover, yam polysaccharides have been shown to have immune-boosting effects and immunomodulatory functions. However, existing research has focused primarily on isolated components rather than evaluating the complete edible form of yam. It is currently unclear whether consuming whole yam has a protective effect on immunosuppressed mice, and what the specific mechanism is. As an important source of food and medicine around the world, exploring the immunomodulatory effects of whole yam and its potential mechanisms has critical scientific and practical value. Therefore, the current research was conducted to systematically investigate the protective effect of yam on immunosuppressed mice infected with influenza virus and its potential mechanisms activating the immune system in immunosuppressed mice.

## 2. Materials and Methods

### 2.1. Plant Material, Chemicals, Kits and Antibodies

Yam was purchased from Qinyang Wild Source Huai Pharmaceutical Co., Ltd. (Qinyang, China), identified by Prof. Hongyan Liu, Shandong University of Traditional Chinese Medicine, China. After shade drying and grinding, the yam powder was stored at 4 °C until use. The voucher specimens were deposited in the Shandong Analysis and Test Center, and all chemicals, including cyclophosphamide (CTX), levamisole hydrochloride (LH) and 2-ethyl butyric acid, were commercially sourced from Sigma-Aldrich (Burlington, VT, USA). TRIzol reagent and enzyme-linked immunosorbent assay (ELISA) kits to quantify antibody and cytokine profiles were bought from Invitrogen (Carlsbad, CA, USA). PrimeScript™ RT reagent Kit with gDNA Eraser and TB Green^®^ Premix Ex Taq™ II were sourced from Takara Biotechnology Co., Ltd. (Shiga, Japan). The following antibodies were used: anti-mouse CD3 (clone 17A2, Biolegend, San Diego, CA, USA), CD8a (clone 53-6.7, Biolegend), B220 (clone RA3-6B2, BD Biosciences, San Jose, CA, USA), and CD4 (clone RM4-5, BD Biosciences).

### 2.2. Scanning Electron Microscopy (SEM)

Observations of yam starch granule morphology were conducted by SEM at an accelerating voltage of 10 kV, with samples first sputter-coated with gold under vacuum [17].

### 2.3. X-Ray Diffraction (XRD) Measurement of Yam Powder Particles

The wide-angle XRD spectra of yam particle samples were obtained using an XRD-EMPYREAN diffractometer (PANalytical B.V., Almelo, The Netherlands). Data were acquired under the following conditions: a 2θ range of 3–50°, a step size of 0.01, a scanning rate of 4°/min, and at room temperature [17].

### 2.4. Determination of the Contents of Chemical Components

The whole yam used in this study was characterized for its major chemical components. Total starch content was determined by the anthrone colorimetric method after acid hydrolysis. Resistant starch was analyzed using the Englyst in vitro digestion method with a GOPOD assay kit (Megazyme, Bray, Co. Wicklow, Ireland). Total polysaccharides were quantified by the phenol–sulfuric acid method. Protein content was measured using a BCA protein assay kit (Solarbio Science & Technology Co., Ltd., Beijing, China). Allantoin and adenosine were quantified by high-performance liquid chromatography (HPLC). Total saponins were determined by the vanillin–glacial acetic acid colorimetric method. All measurements were performed in triplicate, and the results were expressed as means ± SD (mg/g dry weight).

### 2.5. Animal Model of Influenza A Infection

Specific pathogen-free male C57BL/6 mice (21–24 g) were used in this experiment. After one week of acclimatization, the mice were randomly assigned to five groups (*n* = 8): normal control (saline + PBS), PR8 (virus only), CTX + PR8 (CTX + virus), oseltamivir (CTX + virus + oseltamivir) and yam (CTX + virus + 15% whole-yam diet). For the yam-treated group, the mice were fed a diet containing 15% whole yam throughout the experiment, starting from two weeks prior to CTX injection until the end of the experiment, while the other groups received a standard chow diet. To induce immunosuppression, mice in the CTX + PR8, oseltamivir and yam groups received an intraperitoneal injection of CTX (80 mg/kg) once daily for three consecutive days. Mice in the normal control and PR8 groups received an equal volume of sterile saline. One day after the last CTX injection, mice in the PR8, CTX + PR8, oseltamivir and yam groups were anesthetized using a ketamine/xylazine mixture and subsequently inoculated intranasally with 25 μL of influenza virus strain PR8 in sterile phosphate-buffered saline (PBS) [18]. For the oseltamivir group, mice received oral gavage of oseltamivir phosphate (58.5 mg/kg body weight) daily for five consecutive days starting on the day of virus inoculation. Daily monitoring was conducted during the infection period. On day 5 post-infection, the mice were sacrificed. Two independent biological replicates were performed.

### 2.6. Animal Model of Immunosuppression

Specific pathogen-free C57BL/6 mice were purchased from Beijing Vital River Laboratory Animal Technology Co., Ltd (Beijing, China). Male mice aged 7–8 weeks underwent a 7-day acclimation period. The ambient parameters were maintained at 25 ± 2 °C and 60 ± 10% humidity with a 12 h light/dark cycle. Ad libitum access to food and water was provided throughout this study. After acclimation, the mice underwent random allocation to four experimental groups (*n* = 8): normal control, CTX, LH and yam. These groups were fed a standard diet or a 15% yam-supplemented diet (yam group) for 2 weeks, respectively. Then, the normal control group received daily saline injections, while all other groups were administered CTX (80 mg/kg) intraperitoneally over a three-day period to generate an immune injury model. During these 3 days, mice in the LH group underwent oral gavage with LH (5 mg/kg) every day [19]. At the experimental endpoint, mice were euthanized to collect spleen, thymus, colonic tissue, and serum samples.

### 2.7. GPCR Triple-Knockout Mice Experiment

To validate the involvement of SCFA-sensing GPCRs in the immunomodulatory effects observed in yam-treated mice, male GPR41/43/109A triple-knockout mice (7–8 weeks) on a C57BL/6 background were used. Wild-type C57BL/6 mice from the same vendor were used as controls. After 7-day acclimation, mice were randomly assigned to five groups (*n* = 8): normal control, CTX, LH, yam and yam + GPR41/43/109^−/−^. Two independent experiments were performed. The normal control, CTX and LH groups were fed a standard diet, while yam and yam + GPR41/43/109^−/−^ were fed a 15% yam-supplemented diet for 2 weeks. Then, the normal control group received daily saline injections, while all other groups were administered CTX (80 mg/kg) intraperitoneally over a three-day period to generate an immune injury model. During these 3 days, mice in the LH group underwent oral gavage with LH (5 mg/kg) every day [19]. At the experimental endpoint, the mice were euthanized to collect spleen, thymus, colonic tissue, and serum samples. Two independent biological replicates were performed.

### 2.8. Body Weight and Organ Indexes

The daily body weight of the mice was measured. The organ indexes for the spleen and thymus were calculated from their weights and the final body weight, according to the standard formula: index = (organ weight/body weight) × 100.

### 2.9. Flow Cytometric Analysis

Single-cell suspensions from spleen samples were generated by passing the tissue through a 70 μm mesh. The obtained cell suspension was treated with red blood cell lysis buffer for 3 min, followed by centrifugation at 1500 rpm for 5 min at 4 °C. After discarding the supernatant, the resulting pellet was resuspended to obtain lymphocytes. For subsequent assays, 96-well plates were seeded with lymphocytes (1–3 × 10^6^ cells/well). Following a 40 min incubation with antibodies against mouse CD3, B220, CD8 and CD4, lymphocyte subsets including B cells, CD8^+^ and CD4^+^ T cells were analyzed by flow cytometry [20].

### 2.10. Measurement of SCFAs

The contents of acetic, propionic, butyric and valeric acids in feces were detected by gas chromatography–mass spectrometry (GC-MS) according to the previously described method. A 20 mg aliquot of feces was processed by homogenization and 40-fold dilution in ultrapure water with the aid of a microtube homogenizer. Following sonication for 15 min and centrifugation (10,000 rpm, 10 min, and 4 °C), the supernatant was collected. Following the addition of 10 μL of 2-ethyl butyric acid (internal standard) to 100 μL of the supernatant, the mixture was derivatized with PBS and PFBB (44 and 280 μL, respectively) at a temperature of 60 °C for 1.5 h, after which *n*-hexane (200 μL) was added. Then, the samples were subjected to centrifugation at 10,000 rpm for 10 min, and analysis was performed on the upper organic layer using a 6890N-5973N GC-MS (Agilent, Santa Clara, CA, USA) following its transfer to a sample vial. Gas chromatography was performed on an HP-5 capillary column. The injector was set at 250 °C with a 1 μL injection volume and a 30:1 split ratio. The oven temperature program was as follows: hold at 70 °C for 3 min, increase to 130 °C at 10 °C/min, further increase to 280 °C at 30 °C/min, and conclude with a 2 min hold. The MS system operated with the ion source configured to 230 °C and the transfer line at 290 °C. Electron impact (EI) was employed at 70 eV, and the solvent delay was 7 min. Scans were performed using selected ion monitoring (SIM) mode. SCFAs were measured using the corresponding calibration curves [21].

### 2.11. 16S rRNA Sequencing

PCR amplification was performed after genomic DNA extraction from mouse fecal samples and confirmation of its purity using 1.0% agarose gel electrophoresis. Specific primer pairs were employed to amplify the V3-V4 hypervariable region of the bacterial 16S rDNA. Following purification, sequencing of the PCR products was conducted on an Illumina NovaSeq platform (Illumina, Inc., San Diego, CA, USA), which was performed by Suzhou Bionovogene (Suzhou, Jiangsu, China). The proportion of predominant bacteria at multiple taxonomic levels, including the phylum and genus, and all assessments were conducted using the R software 4.6.0. Linear discriminant analysis (LDA) coupled with effect size (LEfSe) was employed to identify and visualize differentially abundant taxa [22].

### 2.12. Histological Evaluation

After collection, tissues (spleen and lung) were PBS-rinsed. Fixation was carried out using 4% neutral paraformaldehyde over 24 h. Subsequent processing included dehydration via an ethanol gradient, paraffin embedding, microtome sectioning, H&E staining (5 min), and mounting with neutral gum. Spleen tissues were examined histologically using a light microscope (Nikon, Tokyo, Japan) [23].

### 2.13. Determination of Serum Antibodies and Cytokine Levels

Quantification of TNF-α, IFN-γ, IL-1β and IL-6 in colonic tissues was conducted using commercial ELISA kits, following the manufacturer’s protocols. Similarly, levels of total IgA, IgG and IgM in serum were measured using ELISA kits [23].

### 2.14. Quantitative Real-Time Polymerase Chain Reaction (qPCR)

Following extraction from colonic tissues with TRIzol reagent, total RNA was assessed for its quantity and purity using a Nanodrop spectrophotometer (Thermo Fisher Scientific, Inc., Waltham, MA, USA). cDNA was synthesized from 1 μg of total RNA using the PrimeScript™ RT reagent Kit with gDNA Eraser. Quantitative real-time PCR was carried out on a Light Cycler480 (Roche, Basel, Switzerland) with TB Green^®^ Premix Ex Taq™ II to determine gene expression levels. All primer pairs utilized in this work were commercially provided by BGI Techsolutions (Beijing Liuhe) Co., Ltd. (Beijing, China), and their sequences are detailed in Table 1. The relative mRNA expression levels of occludin, claudin, ZO-1, TNF-α, IFN-γ, IL-1β, IL-6 and M were quantified using the 2^−∆∆Ct^ method, with Gapdh for normalization [24].

### 2.15. RNA-Sequencing Analysis

Library preparation and gene sequencing were completed by Wekemo Tech Group Co., Ltd. (Shenzhen, China). For the construction of cDNA libraries intended for RNA sequencing, total RNA was isolated from collected thymus samples with TRIzol^®^ Reagent (Invitrogen, Carlsbad, CA, USA), as directed by the manufacturer’s protocol. RNA quality control involved integrity analysis on an Agilent 5300 Bioanalyser (Agilent) and concentration measurement via a NanoDrop2000 (Thermo Fisher Scientific, Inc., Waltham, MA, USA). Paired-end sequencing (150 bp) was conducted on a DNBSEQ-T7 (MGI Tech Co., Ltd., Shenzhen, China). In this study, genes meeting the criteria of |log_2_(FoldChange)| > 1 with a *p*-value of < 0.05 were defined as differentially expressed genes (DEGs). GO and KEGG enrichment analyses were carried out to obtain functional annotation and biological interpretation of these DEGs [25].

### 2.16. Statistical Analysis

The data in this study are expressed as means ± SD. Statistical significance was determined by Student’s *t*-test using GraphPad Prism 9, and the significance levels were set at *p* < 0.05.

## 3. Results

### 3.1. Physicochemical Properties and Bioactive Components of Yam Powder

**Figure 1:**
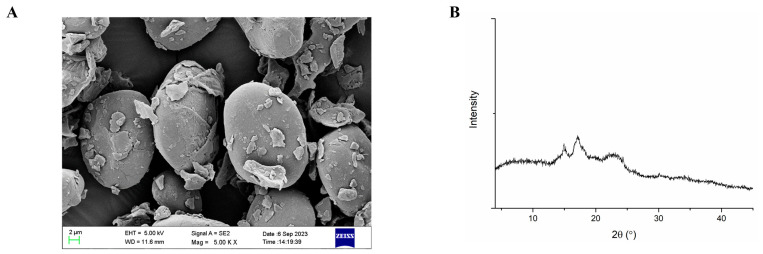
Granule morphology and crystalline characteristics of yam powder particles. (**A**) SEM micrographs showing surface microstructure of yam powder particles. (**B**) Wide-angle XRD patterns of yam powder particles.

The morphologies of yam powder particles are shown in Figure 1A. The yam particles were characterized by their round/oval shape and rough surface topography. The crystalline characteristics of the yam powder sample were determined by XRD, as shown in Figure 1B. According to the crystallization properties and the distinct XRD signatures, starch from diverse botanical sources could be categorized into types A, B, C and V. The yam exhibited an A-type crystal structure with peaks located at 15°, 17°, and 23°.

The chemical composition of the yam powder was further analyzed (Table 2). Starch was the most abundant component (694.30 ± 3.383 mg/g), followed by resistant starch (245.00 ± 2.887 mg/g). The contents of total polysaccharides, protein, allantoin, saponins, and adenosine were 70.92 ± 0.176, 43.60 ± 0.119, 24.43 ± 0.747, 1.14 ± 0.029, and 0.53 ± 0.008 mg/g, respectively.

### 3.2. Yam Protected Immunodeficient Mice Against Influenza Virus Infection

**Figure 2:**
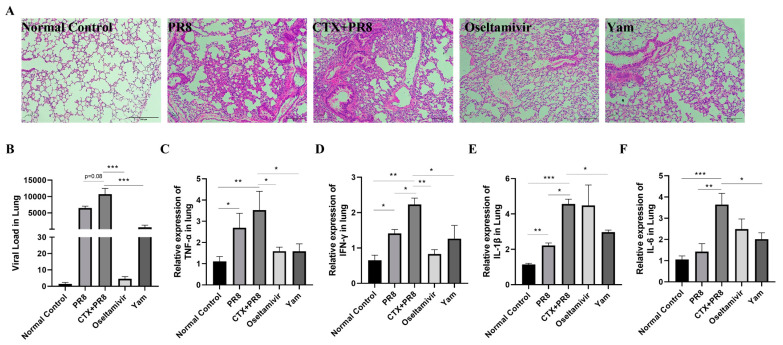
Yam protected immunodeficient mice against influenza virus infection. (**A**) Representative images of HE-stained lung sections. Yam significantly reduced the viral load (**B**) and expression levels of TNF-α (**C**), IFN-γ (**D**), IL-1β (**E**) and IL-6 (**F**) in lung tissues. Values are presented as mean SD ± SEM. * *p* < 0.05, ** *p* < 0.01 and *** *p* < 0.001.

As aforementioned, immunodeficiency increases susceptibility to infection. Therefore, a mouse model of influenza infection was employed to evaluate the efficacy of yam. Histological analysis of lung tissue revealed a normal structure in the normal control group, whereas the CTX + PR8 group exhibited obvious edematous changes in the interstitium, accumulation of inflammatory cells and alveolar enlargement. However, a significant alleviation of interstitial edema, reduction in inflammatory infiltration and diminished alveolar widening were observed in the yam group (Figure 2A). Moreover, the CTX + PR8 group exhibited a greater viral load compared with the PR8 group, indicating that CTX-induced immunocompromised mice are more susceptible to influenza virus infection, while yam significantly reduced the viral load (Figure 2B). Consistent with the above findings, the expression levels of inflammatory cytokines (TNF-α, IFN-γ, IL-1β and IL-6) were significantly upregulated in the CTX + PR8 group but downregulated with yam supplementation (Figure 2C–F).

### 3.3. Effects of Yam Supplementation on Preventing CTX-Induced Immunodeficiency in Mice

**Figure 3:**
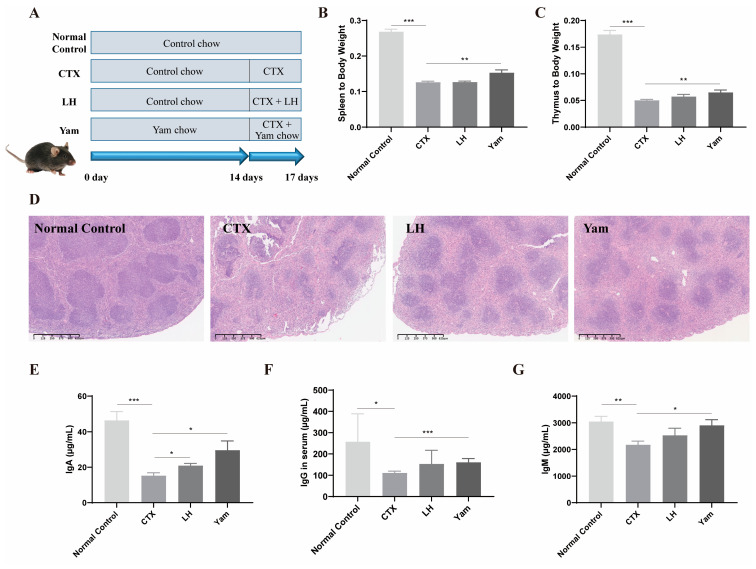
Protective effect of yam against CTX-induced damage in mice. The animal experimental protocol (**A**) and effect of yam on the spleen index (**B**) and thymus index (**C**). Representative HE-staining images of spleen sections (**D**). Yam significantly upregulated the expression of IgA (**E**), IgG (**F**) and IgM (**G**) in serum. Values are presented as mean SD ± SEM. * *p* < 0.05, ** *p* < 0.01 and *** *p* < 0.001.

The overall health status and alterations in mice weight before and after CTX injection were monitored to determine whether the mouse model was successfully established. Prior to CTX injection, mice in each group had bright eyes, glossy fur and quick movements. After the injection of CTX, the mice became weak, their fur was dull and sparse, and their weight began to drop sharply, which was significantly different from before injection, indicating that the modeling was successful. The immune organ index primarily serves to indicate the body’s immune response level, while the weight of the immune organ can be utilized to measure the growth and development of the organ. The CTX group exhibited a significant reduction in spleen and thymus indices compared with the normal control group. However, these indices were significantly improved in the yam group, preliminarily suggesting that yam has a protective effect on immunodeficiency (Figure 3B,C).

In addition, we assessed the impact of yam on immune competence in immunosuppressed mice following CTX administration by examining the morphology of spleen tissue and the levels of immunoglobulins. HE-staining analysis of the spleen is shown in Figure 3D. In the normal control group, the morphology and number of germinal centers were normal, and the splenic cells showed a compact and organized architecture with clearly discernible nuclei. Relative to the normal control group, the CTX group presented with dispersed germinal centers as well as a significant decrease in the number of lymphocytes. In contrast with the CTX group, the yam group exhibited clearer germinal centers and a more compact lymphocyte arrangement.

Immunoglobulins are a class of polyclonal antibodies, including IgA, IgG, IgM and IgE, which are essential for the immune response as they specifically recognize and neutralize pathogens, thus playing a key role in protecting the body from infection. To assess humoral immune status, the concentrations of serum immunoglobulins (IgA, IgG and IgM) were quantified. As shown in Figure 3E–G, CTX notably reduced serum levels of IgA, IgG and IgM. In contrast, yam administration resulted in markedly higher levels of these immunoglobulins, indicating that yam treatment had a positive effect on immunity in immunosuppressed mice.

**Figure 4:**
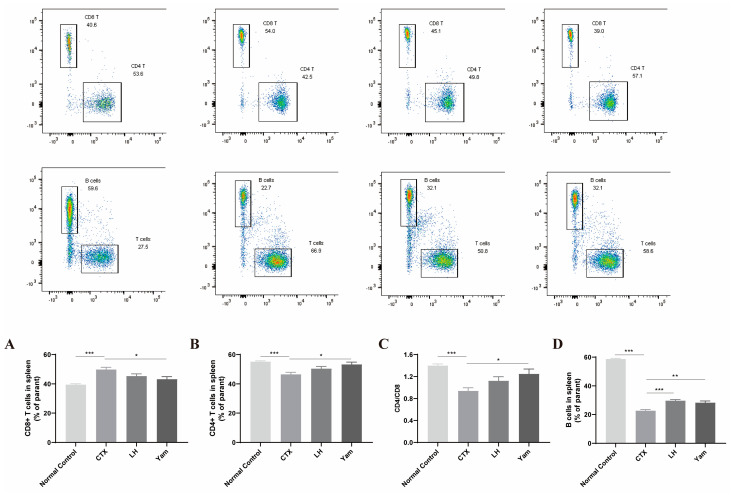
Flow cytometric analysis of splenic lymphocyte subpopulations. Splenic frequencies of CD4^+^ T cells (**A**) and CD8^+^ T cells (**B**) along with ratio of CD4/CD8 (**C**) and frequency of B cells (**D**) in the spleen. Values are presented as mean SD ± SEM. * *p* < 0.05, ** *p* < 0.01 and *** *p* < 0.001.

We further investigated whether yam affects T lymphocyte and B lymphocyte subsets in the spleen. The results revealed that yam prevented CD8^+^ T cells from increasing and CD4^+^ T cells from decreasing. In comparison with the normal control group, a significant increase in the CD8^+^ T cell population and a marked decrease in CD4^+^ T cell and B cell populations were observed in the CTX group by flow cytometry. However, the frequencies of these lymphocytes in the yam group were significantly restored relative to the CTX group (Figure 4). It also showed that yam significantly increased the ratio of CD4^+^/CD8^+^. These results collectively indicate that yam has a positive regulatory effect on the body’s immunity.

### 3.4. Effects of Yam on Intestinal Barrier Function in Immunosuppressed Mice

**Figure 5:**
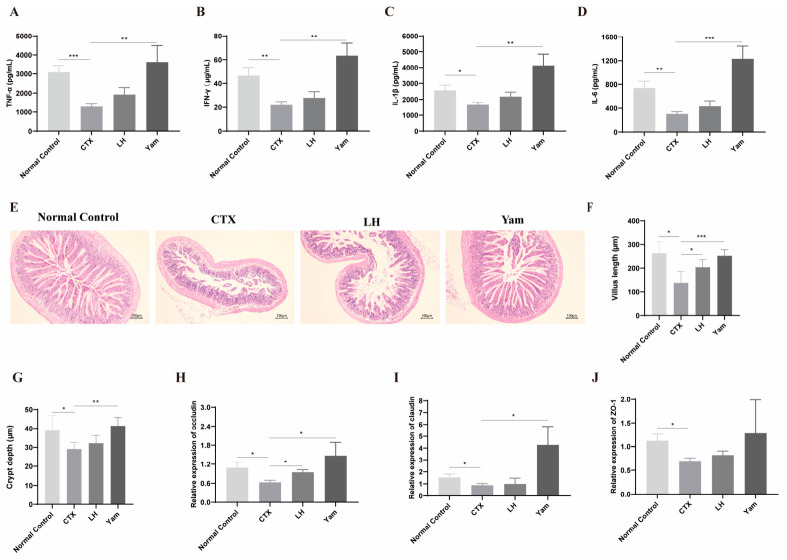
Effects of yam on colonic cytokine factors and intestinal barrier in immunosuppressed mice. The colonic levels of TNF-α (**A**), IFN-γ (**B**), IL-1β (**C**) and IL-6 (**D**) were determined using ELISA. Representative HE-staining images of colon sections (**E**). The villus length (**F**) and crypt depth (**G**) were measured. The expression levels of occludin (**H**), claudin (**I**) and ZO-1 (**J**) were detected using qPCR. Values are presented as mean SD ± SEM. * *p* < 0.05, ** *p* < 0.01 and *** *p* < 0.001.

Cytokines are vital proteins that can regulate the functions of T helper cells. In this study, the expression levels of TNF-α, IFN-γ, IL-1β and IL-6 in the colon were determined. As shown in Figure 5A–D, compared with the normal control group, CTX administration significantly reduced the expression levels of TNF-α, IFN-γ, IL-1β and IL-6. In contrast, yam treatment restored their expression.

As evidenced in Figure 5E, the extent of intestinal barrier damage was greater in the CTX group than in the normal control group, manifested as aggravated inflammation and destruction of intestinal structure. In contrast, yam supplementation significantly reduced the inflammation and damage in the intestinal barrier, as evidenced by improvements in villus length and crypt depth (Figure 5F,G). In addition, the level of intestinal permeability reflects the functional state of the mucosal barrier and is an essential predictor of potential bacterial translocation. To investigate the protective role of yam on the mucosal barrier, intestinal permeability was measured. According to Figure 5H–J, CTX administration impaired the intestinal mucosal barrier, as indicated by a significant rise in permeability. This impairment was associated with downregulated tight-junction proteins, namely, occludin, claudin and ZO-1. However, treatment with yam significantly increased the transcript levels of occludin, claudin and ZO-1 genes. Combined with the observed improvements in intestinal villi and crypt architecture, our data provided evidence that yam enhanced barrier integrity and maintained gut homeostasis.

### 3.5. Effects of Yam on SCFA Production and Gut Microbiota Dysbiosis

**Figure 6:**
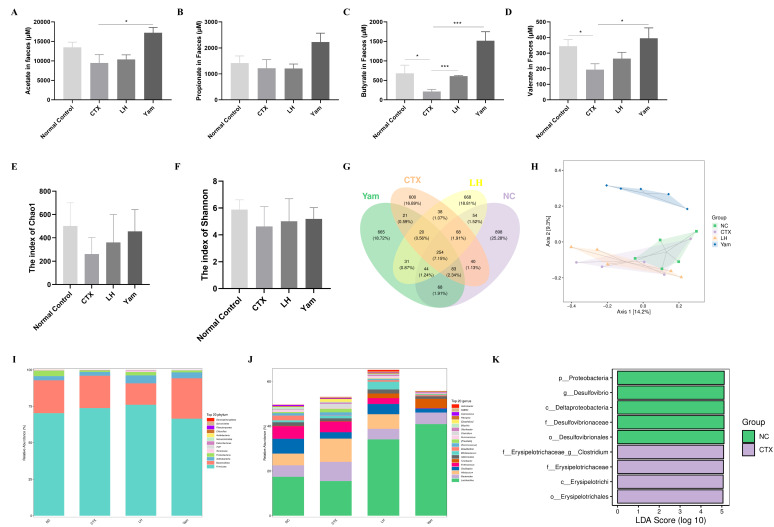
Effects of yam on the production of SCFAs in feces and gut microbiota dysbiosis. Concentration of acetate, propionate, butyrate and valerate (**A**–**D**). Chao1 and Shannon indices (**E**,**F**). Venn diagram (**G**). PCoA score plot (**H**). The relative abundance of gut microbiota at the phylum level and the genus level (**I**,**J**). LEfSe was conducted to determine abundance differences: LDA score plot (**K**). Values are presented as mean SD ± SEM. * *p* < 0.05 and *** *p* < 0.001.

To investigate the functional consequences of yam supplementation on the metabolomic profile of the intestinal microbiota, the concentrations of SCFAs in fecal samples were determined. The results in Figure 6A–D show that relative to the normal control group, acetate, propionate, butyrate and valerate were all reduced in the CTX group, with butyrate and valerate exhibiting the most pronounced decreases. Yam intake, however, significantly elevated fecal concentrations of acetate, butyrate and valerate relative to the CTX group.

Furthermore, the composition of the gut microbiota was detected. Alpha diversity reflects the richness and diversity of the gut microbial community. As shown in Figure 6E,F, the richness of the microbial community decreased in the CTX group after administration, while that in the yam group increased. According to the Shannon index, a decrease in microbial diversity was observed in the CTX group, contrasting with an increase in the yam group. The Venn diagram revealed varying OTU counts among the groups: the normal control group had the highest (1509), followed by the yam (1186), LH (1177) and CTX (1124) groups (Figure 6G). Then, beta diversity was analyzed using PCoA to visualize differences in microbial community structure. The PCoA plot showed that the yam group formed a distinct cluster, separate from the overlapping clusters of the normal control, CTX, and LH groups (Figure 6H). According to taxonomic analysis, it was found that the gut microbiota in all samples was mainly composed of four major phyla: Bacteroidota, Proteobacteria, Firmicutes and Verrucomicrobiota. In immunocompromised mice, Firmicutes increased, elevating the Firmicutes/Bacteroidetes (F/B) ratio (3.32), whereas the F/B ratio (2.40) was greatly decreased in the yam group (Figure 6I). Moreover, at the genus level, Allobaculum became more abundant, and Lactobacillus became less abundant. These changes in the composition of bacteria, which are key contributors to SCFA generation, especially Allobaculum, were both restored by yam treatment (Figure 6J). Additionally, according to the LEfSe analysis, it was confirmed that yam treatment reduced the CTX-induced increase in the Clostridium population (Figure 6K). All of these findings indicate that yam may promote the immune response by regulating the gut microbiota and SCFA production.

### 3.6. Genetic Deletion of GPR41, GPR43 and GPR109A Was Associated with Impaired the Effects of Yam

SCFAs such as acetate play an important role in influencing immunity. It has been suggested that SCFAs exert their effects through GPCR signaling. Given the beneficial effects of yam on immunodeficiency, we examined the potential mediation by key metabolite-sensing GPCRs: GPR41, GPR43 and GPR109A using GPR41/43/109A^−/−^ mice.

**Figure 7:**
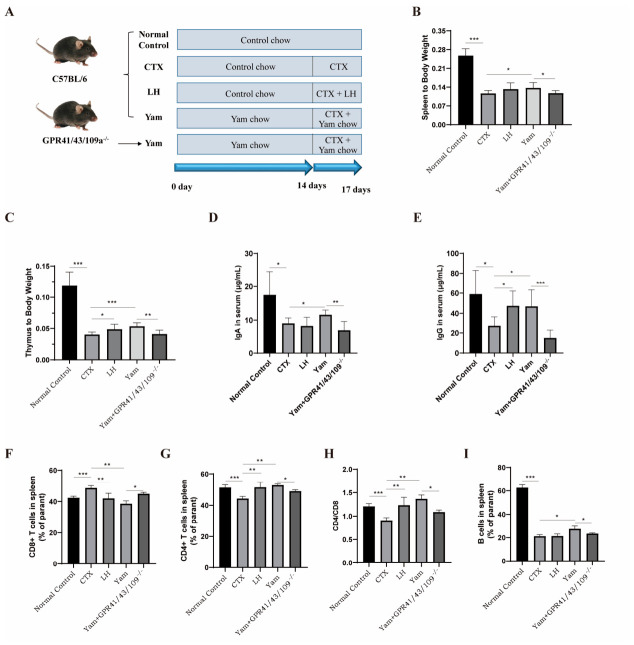
GPR41, 43 and 109A were involved in the protective process of yam. The animal experimental protocol (**A**) and the spleen index (**B**), thymus index (**C**), and expression levels of IgA (**D**) and IgG (**E**) in serum were determined in wild-type and GPR41/43/109A^−/−^ mice that did or did not receive yam treatment. The flow cytometric profiling of splenic lymphocytes included the frequencies of CD8^+^ T cells (**F**) and CD4^+^ T cells (**G**), and the ratio of CD4/CD8 (**H**) and the splenic frequency of B cells (**I**) were analyzed. Values are presented as mean SD ± SEM. * *p* < 0.05, ** *p* < 0.01 and *** *p* < 0.001.

As illustrated in Figure 7, administration of a yam diet to wild-type mice ameliorated clinical symptoms, including increased spleen and thymus indices and elevated expression levels of IgA and IgG in serum, whereas GPR41/43/109A^−/−^ mice fed a yam diet showed suppression of the protective effects of yam. To further define the involvement of GPCRs, we assessed whether GPCR signaling was required for the protective effects of yam in immunodeficient mice. The results showed that in GPR41/43/109A^−/−^ mice, the yam diet failed to induce a reduction in CD8^+^ T cells or an increase in CD4^+^ T cells, the CD4^+^/CD8^+^ ratio, or B cell levels.

### 3.7. Transcriptome Analysis

**Figure 8:**
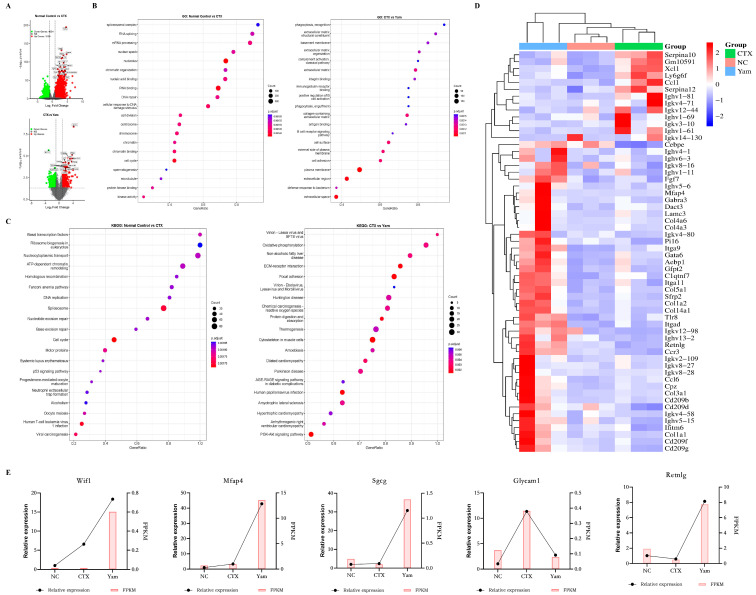
Transcriptomic analysis of effects of yam on CTX-induced immunodeficiency. Volcano plot of the DEGs (**A**). Functional enrichment of the overlapping DEGs is presented, including GO terms (**B**) and the top-20 enriched KEGG pathways (**C**). Heatmap of immune-related genes (**D**) and validation of RNA-seq results using qPCR (**E**).

This study used RNA-sequencing technology to analyze the gene expression profile of thymus tissue, aiming to explore the mechanisms behind the protective effects of yam. Differentially expressed gene analysis was conducted to identify DEGs between treatment groups to help elucidate the potential protective mechanisms involved. The transcriptomic profiles of thymus samples from the three experimental groups were compared using the DESeq package in R, and the number of DEGs was visualized using a volcano plot (Figure 8A). A total of 7309 DEGs were detected after CTX treatment, of which 4024 genes were downregulated, and 3105 genes were upregulated. Comparison of the yam group with the CTX group revealed 548 DEGs, comprising 262 downregulated genes and 286 upregulated genes. GO analysis showed that CTX induced intracellular toxicity, mainly disrupting essential nuclear processes including genome maintenance and cell proliferation, which is highly consistent with the known mechanism of CTX as a DNA-damaging alkylating agent. After treatment with yam, the enrichment profile showed significant signals in extracellular matrix organization, a classic hallmark of tissue remodeling and wound healing, suggesting that yam may promote the reconstruction of CTX-damaged tissue architecture. Furthermore, the significant enrichment of immune-related functions (B cell activation, complement cascade and antigen binding) indicated that yam contributed to the reversal of the immunosuppressive effects of CTX (Figure 8B). KEGG pathway analysis was carried out to provide a high-level view of the biological pathways disrupted by CTX and subsequently modulated by yam treatment. The results showed that CTX induced severe genotoxic stress, manifested by significant enrichment of DNA damage response pathways, including nucleotide excision repair, the p53 signaling pathway and DNA replication. Instead, yam treatment promoted a shift in tissue toward restoration and remodeling. This was indicated by the presence of pathways related to extracellular matrix organization, such as ECM–receptor interaction, focal adhesion, and energy metabolism, such as oxidative phosphorylation. Furthermore, the enrichment of immune-related pathways (generally annotated under viral infection terms) suggested that yam could also ameliorate CTX-induced immunosuppression and modulate inflammatory responses during the repair process (Figure 8C). The involvement of the PI3K-Akt signaling pathway, a central pathway regulating cellular homeostasis (e.g., survival and growth), further supports the protective mechanism of yam. Overall, these results strongly support the narrative derived from the GO analysis. In addition, the DEGs related to immune response were displayed in the form of a heat map (Figure 8D). Among them, the relative expression levels of Wif1, Mfap4, Sgcg, Glycam1 and Retnlg were determined by qPCR. The results were consistent with the changing trends in gene expression levels obtained by transcriptome sequencing, indicating that the transcriptome data were highly reliable (Figure 8E).

## 4. Discussion

A state of immunosuppression, common in both animals and humans, arises from a variety of factors, including illness, trauma, stress, toxins or side effects of therapeutic drugs [1,26]. Weakened immune function increases susceptibility to infection and disease, making it difficult for the body to mount an effective defense against pathogens [2]. Regulation of the immune system constitutes a key defense against diseases, especially those caused by viruses and pathogens. Therefore, the development of novel immune enhancers has become crucial [27]. To contribute to this effort, this study focused on evaluating the effects of yam on immunodeficiency. The results showed that yam significantly alleviated the immunosuppressive effects induced by CTX in mice, as demonstrated by the recovery of the spleen and thymus, which indirectly represent immune function.

T lymphocyte subsets, which are vital components in the immune response, mainly comprise CD8^+^ and CD4^+^ T cells. As cytotoxic lymphocytes, CD8^+^ T cells serve as important mediators in anti-tumor immunity, possessing the capacity to kill cancer cells, whereas CD4^+^ T cells provide crucial support by assisting B cells in secreting antibodies and coordinating the adaptive immune response via the secretion of pleiotropic cytokines involved in chemotaxis, inflammatory signaling, and the maintenance of immune homeostasis [28,29]. Given their importance, we also investigated the impact of yam on the proportions of CD8^+^ and CD4^+^ T cells, along with the splenic CD4^+^/CD8^+^ ratio. The results revealed that yam mitigated the increase in CD8^+^ T cells and the decrease in CD4^+^ T cells. Moreover, a major mechanism by which CD4^+^ T cells enhance immune reactions is through the secretion of key inflammatory mediators (e.g., TNF-α, IFN-γ, IL-1β and IL-6). As a pivotal immune-related cytokine, TNF-α functions as a key mediator of innate immunity and a potential indicator of immune function [30]. Acting as a multifunctional cytokine, IFN-γ serves as a key orchestrator of various immune responses, such as enhancing antiviral activity, activating macrophages, and modulating cell apoptosis and proliferation [31]. IL-1β is a key component of the IL-1 family and has the ability to activate various complement components, as well as several cytokines, including TNF-α and even itself. IL-6 acts as a broad-acting cytokine with diverse functions in maintaining immune homeostasis and controlling the inflammatory cascade [32]. In this study, yam restored the production of TNF-α, IFN-γ, IL-1β and IL-6 in colonic tissue, potentially promoting immune responses in immunosuppressed mice. It should be noted that the colonic cytokine data were obtained from the immunosuppression model (CTX only), whereas the pulmonary cytokine data were from the influenza infection model (CTX + PR8 challenge).

Adaptive immunity relies heavily on B cells. Upon antigen exposure, these cells get activated and subsequently differentiate into two key subsets: plasma cells, which are responsible for secreting antibodies, and memory B cells, which provide long-term immunological memory by rapidly responding upon subsequent encounters with the identical antigen [33,34]. The results indicated that yam significantly inhibited the reduction in B cells caused by CTX. In line with these observations, the current study demonstrated that yam enhanced the production of key serum immunoglobulins. These immunoglobulins are essential components in the humoral immune responses, which is a major way B cells contribute to immune defense. These results, combined, suggested that yam has the ability to enhance immunity.

While IgG antibodies serve as the main effectors of influenza protection, multiple lymphocyte populations, namely, CD4^+^ T cells, CD8^+^ T cells and B cells, act individually and synergistically to provide antiviral immunity [35]. Complications or eventual death from influenza virus infection are often associated with excessive release of inflammatory mediators (TNF-α, IFN-γ, IL-1β and IL-6), a phenomenon known as a “cytokine storm” [36]. Here, we observed that yam treatment alleviated the pathological phenotype in immunocompromised mice with influenza virus infection, as manifested by reduced viral load and attenuated inflammatory response in the lungs, further verifying the potential of yam to enhance immunity.

Furthermore, the intestinal epithelial barrier, a single-cell-thick layer lining the intestine, acts as a vital interface that offers primary defense against external threats. Despite this defensive role, it simultaneously supports a symbiotic interaction with the microbiome. This dual function is critically dependent on the integrity provided by tight-junction proteins, such as occludin, claudin and ZO [37]. Therefore, any impairment of the barrier’s structure represents a key event in initiating an uncontrolled immune response within the intestinal microenvironment or leads to unrestricted growth of the microbiota. Thus, the preservation of intestinal barrier integrity underpins key physiological functions of the human body [38]. The results of this study showed that yam significantly increased the expression levels of occludin, claudin and ZO-1. Combined with the observed improvements in intestinal villi and crypt architecture, these findings suggest that yam treatment can enhance intestinal barrier integrity and maintain gut homeostasis.

In fact, microbial metabolites such as SCFAs are now considered important messengers in host–microbiota crosstalk. Their function in maintaining gut homeostasis and overall health makes them indispensable. SCFAs are absorbed and serve as a primary energy source in the gut, and the SCFAs create an acidic environment that suppresses pathogens and modulates the composition of the gut microbiota [12,13]. Based on the observed efficacy of yam in improving the effect on intestinal immune response and intestinal barrier function through tight-junction proteins’ upregulation, we hypothesized that these effects might be mediated by shifts in the gut microbiota and its metabolic output, particularly SCFAs. This hypothesis is supported by a growing body of evidence on plant-derived polysaccharides. As reviewed recently, dietary polysaccharides enhance the abundance of beneficial intestinal microbes and increase SCFA production, thereby improving gut health and supporting immune system development [39,40].

GPCRs are currently thought to mediate the majority of nutrient sensing in the gastrointestinal tract and serve as a biochemical bridge connecting metabolic processes and immune regulation [41]. GPR43 is activated by SCFAs (acetate, propionate, butyrate and valerate) and participates in multiple immune processes such as Treg biology and neutrophil chemotaxis [41,42,43]. Given that GPR41 functions predominantly as the receptor for acetate and propionate (with secondary activation by butyrate and valerate), its signaling affects the development of T helper cells and airway eosinophilic inflammation in response to house dust mite challenge [44,45]. GPR109A was originally characterized as a niacin receptor and was later found to exhibit higher physiological relevance through butyrate binding. Our study investigated the underlying protective mechanisms by using GPR41/43/109A^−/−^ mice and RNA sequencing technology, providing novel insights into the specific pathways involved.

GPR41, GPR43 and GPR109A, involved in the current study, recognize and interact with a variety of metabolites from dietary sources, especially SCFAs, and transmit key signals for maintaining normal immune and metabolic activities. What distinguishes these receptors is their ability to transmit anti-inflammatory signals, control metabolic processes and maintain intestinal homeostasis. Expression of these molecular sensors is found in a broad spectrum of immune cells, ranging from innate cells like neutrophils and dendritic cells to adaptive components such as T and B cells [42,43,46]. Beyond the immune system, the intestinal lining serves as another major location for these receptors, likely related to their function in preserving epithelial barrier function. These receptors function as a principal mechanism mediating the gut microbiota’s impact on the immune system. The results of transcriptome analysis suggested that yam could activate GPR41 and GPR43 on various cell types by SCFAs to promote glucose uptake and enhance mitochondrial function because simultaneous activation of oxidative phosphorylation and the thermogenesis pathway suggested a heightened demand for ATP to fuel the energetically costly processes of tissue repair and immune activation [47,48]. Signaling through GPR41, GPR43 and GPR109A in immunocytes helped restore the immune imbalance induced. It was supported by the enrichment of immune-related terms, which are often annotated under viral infection pathways, suggesting a reinstatement of immune competence. Furthermore, activation of GPR41, GPR43 and GPR109A may directly regulate gene expression related to cell migration, proliferation and ECM synthesis. Furthermore, the observed attenuation of yam’s protective benefit in GPR41/43/109A^−/−^ mice suggested that SCFA-mediated signaling through GPCRs played a crucial role in yam’s immunomodulatory actions. This aligns with the known functions of SCFAs in regulating immune responses and maintaining gut homeostasis. Taken together, yam likely mediated its activity using SCFAs to enhance immunity by activating GPR41, GPR43 and GPR109A, which, in turn, orchestrate the observed metabolic, immune and reparative responses.

The multiple benefits observed in this study—from gut microbiota remodeling and SCFA production to enhanced systemic immunity—are likely attributable to the synergistic effects of various bioactive components in whole yam, such as polysaccharides, proteins, and dietary fiber. Several studies have directly investigated the effects of yam-derived components in the CTX-induced immunosuppression model. Yam polysaccharides have been shown to contribute to immune enhancement. Studies have demonstrated that yam polysaccharides increase the thymus index, serum immunoglobulins and cytokines in broilers [49]. Moreover, Chinese yam peel extracts (rich in polysaccharides) were shown to reverse CTX-induced decreases in immune organ indices, repair the intestinal barrier and modulate the gut microbiota. Yam protein also ameliorated CTX-induced intestinal immunosuppression by regulating the gut microbiota and altering metabolic pathways, such as tryptophan metabolism [50]. Additionally, yam protein restored immunoglobulins and cytokines in CTX-treated mice. Furthermore, yam protein was found to protect against CTX-induced testicular injury by suppressing the TLR4/MyD88/NF-κB pathway and upregulating tight-junction proteins [51,52]. These findings suggest that yam protein may serve as a key immunomodulatory component. Based on these findings, we propose that the immunoprotective effect of whole yam arises from multicomponent synergy. Yam polysaccharides and resistant starch serve as fermentable substrates for the gut microbiota, promoting SCFA production, the central link to GPCR-mediated immune activation. Yam protein contributes through the TLR4/MyD88/NF-κB signaling pathway and tryptophan metabolism, which may synergize with SCFA-GPCR signaling. Allantoin, saponins and adenosine may provide additional gut microbiota regulation support [53,54]. Future studies employing component isolation and reconstitution approaches are warranted to delineate the relative contribution of each component.

This synergistic effect may be difficult to replicate with isolated components, highlighting the value of studying whole foods in edible forms. Our method of using whole yam can more realistically simulate its regular dietary intake, thereby providing translational application value for the development of yam-based functional foods or dietary supplements aimed at enhancing immunity. We acknowledge that the complexity of the whole food matrix presents a challenge to accurately attributing effects to specific compounds. However, the clear mechanistic pathway elucidated here—the gut microbiota–SCFA–GPR41/43/109A axis—provides a strategic starting point for future research.

We acknowledge that the triple-knockout experiment did not distinguish the individual contributions of GPR41, GPR43 and GPR109A. Single-receptor knockout studies would be needed to determine whether a single receptor is sufficient or whether all three are required for the yam-mediated immune effect. Nevertheless, given the partially overlapping ligand specificities and functional redundancy among these receptors, the triple-knockout approach provides direct genetic evidence that the SCFA–GPCR axis as a whole is involved in the immunoprotective effect of whole yam [46]. In addition, we acknowledge that survival curves and body weight changes were not included in the current study. Future PR8 challenge studies should include these endpoints to provide a more complete assessment of yam’s protective effects.

## 5. Conclusions

In summary, our findings suggest that dietary supplementation with whole yam may enhance the resistance of immunocompromised mice to influenza virus. The underlying mechanism may involve yam-mediated gut microbiota regulation, thereby increasing SCFA production, enhancing the gut barrier, and activating T and B cell responses via the GPCR signaling pathway, jointly promoting antiviral defense. Collectively, our study provides strong evidence supporting the use of whole yam as an easily accessible food-based dietary strategy to enhance immunity by modulating the gut ecosystem, particularly for individuals with compromised immune function.

## Figures and Tables

**Table 1:** Primers used in qPCR experiments.

Gene	Forward	Reverse
Gapdh	CATCACTGCCACCCAGAAGACTG	ATGCCAGTGAGCTTCCCGTTCAG
occludin	TGGCAAGCGATCATACCCAGAG	CTGCCTGAAGTCATCCACACTC
claudin	GGACTGTGGATGTCCTGCGTTT	GCCAATTACCATCAAGGCTCGG
ZO-1	GTTGGTACGGTGCCCTGAAAGA	GCTGACAGGTAGGACAGACGAT
TNF-α	ACGTGGAACTGGCAGAAGAGG	TGAGAAGAGGCTGAGACATAGGC
IFN-γ	GGAACTGGCAAAAGGATGGTGAC	TGACGCTTATGTTGTTGCTGATGG
IL-1β	TCGCAGCAGCACATCAACAAG	TCCACGGGAAAGACACAGGTAG
IL-6	CGGAGAGGAGACTTCACAGAGG	TTCCACGATTTCCCAGAGAACATG
M	CTGAGAAGCAGGTACTGGGC	CTGCATTGTCTCCGAAGAAAT

**Table 2:** Contents of chemical composition of the whole yam.

Composition	Content (mg/g Dry Weight)	Method
Starch	694.30 ± 3.383	Anthrone colorimetric method after acid hydrolysis
Resistant starch	245.00 ± 2.887	Englyst in vitro digestion method with GOPOD assay
Total polysaccharide	70.92 ± 0.176	Phenol–sulfuric acid method
Protein	43.60 ± 0.119	BCA protein assay kit
Allantoin	24.43 ± 0.747	HPLC
Total saponin	1.14 ± 0.029	Vanillin–glacial acetic acid colorimetric method
Adenosine	0.53 ± 0.008	HPLC

## Data Availability

The original contributions presented in the study are included in the article files; further inquiries can be directed to the corresponding authors upon reasonable request.

## Abbreviations

CTX, cyclophosphamide; SCFAs, short-chain fatty acids; LH, levamisole hydrochloride; ELISA, enzyme-linked immunosorbent assay; SEM, scanning electron microscopy; XRD, X-ray diffraction; HPLC, high-performance liquid chromatography; GC-MS, gas chromatography–mass spectrometry; LDA, linear discriminant analysis; qPCR, quantitative real-time polymerase chain reaction; DEGs, differentially expressed genes.

## References

[1] Fali T., Vallet H., Sauce D. (2018). Impact of stress on aged immune system compartments: Overview from fundamental to clinical data. Exp. Gerontol..

[2] Lokken-Toyli K.L., Diaz-Ochoa V.E., Camacho L., Stull-Lane A.R., Van Hecke A.E.R., Mooney J.P., Muñoz A.D., Walker G.T., Hampel D., Jiang X.W. (2024). Vitamin A deficiency impairs neutrophil-mediated control of Salmonella via SLC11A1 in mice. Nat. Microbiol..

[3] Fukuyama S., Kawaoka Y. (2011). The pathogenesis of influenza virus infections: The contributions of virus and host factors. Curr. Opin. Immunol..

[4] Javanian M., Barary M., Ghebrehewet S., Koppolu V., Vasigala V., Ebrahimpour S. (2021). A brief review of influenza virus infection. J. Med. Virol..

[5] Hu J., Chen J., Ma L., Hou Q., Zhang Y., Kong X., Huang X., Tang Z., Wei H., Wang X. (2024). Characterizing core microbiota and regulatory functions of the pig gut microbiome. ISME J..

[6] Li Y., Dong J., Xiao H., Zhang S., Wang B., Cui M., Fan S. (2020). Gut commensal derived-valeric acid protects against radiation injuries. Gut Microbes.

[7] Markonda L., Henderson W.A. (2026). Diet and Microbiota–Gut–Brain Axis: A Novel Nutritional Therapy. Nutrients.

[8] Beukema M., Faas M.M., de Vos P. (2020). The effects of different dietary fiber pectin structures on the gastrointestinal immune barrier: Impact via gut microbiota and direct effects on immune cells. Exp. Mol. Med..

[9] Asayesh M., Nazarzadeh A., Jamshidi S., Keramat S., Ryszkiel I., Stanek A. (2026). Modulation of Gut Microbiota Through Dietary Fibers to Enhance Regulatory T Cell-Based Immunotherapy in GVHD Following Hematopoietic Stem Cell Transplantation. Nutrients.

[10] Yan W., Luo J., Yu Z., Xu B. (2024). A critical review on intestinal mucosal barrier protection effects of dietary polysaccharides. Food Funct..

[11] Zhang C., Pi X., Li X., Huo J., Wang W. (2024). Edible herbal source-derived polysaccharides as potential prebiotics: Composition, structure, gut microbiota regulation, and its related health effects. Food Chem..

[12] Martin-Gallausiaux C., Marinelli L., Blottière H.M., Larraufie P., Lapaque N. (2021). SCFA: Mechanisms and functional importance in the gut. Proc. Nutr. Soc..

[13] Morrison D.J., Preston T. (2016). Formation of short chain fatty acids by the gut microbiota and their impact on human metabolism. Gut Microbes.

[14] Padhan B., Panda D. (2020). Potential of Neglected and Underutilized Yams (*Dioscorea* spp.) for Improving Nutritional Security and Health Benefits. Front. Pharmacol..

[15] Feng Q., Lin J., Niu Z., Wu T., Shen Q., Hou D., Zhou S. (2024). A Comparative Analysis between Whole Chinese Yam and Peeled Chinese Yam: Their Hypolipidemic Effects via Modulation of Gut Microbiome in High-Fat Diet-Fed Mice. Nutrients.

[16] Zhang N., Liang T., Jin Q., Shen C., Zhang Y., Jing P. (2019). Chinese yam (*Dioscorea opposita* Thunb.) alleviates antibiotic-associated diarrhea, modifies intestinal microbiota, and increases the level of short-chain fatty acids in mice. Food Res. Int..

[17] Song Y., Qu X., Guo M., Hu Q., Mu Y., Hao N., Wei Y., Wang Q., Mackay C.R. (2023). Indole acetylated high-amylose maize starch: Synthesis, characterization and application for amelioration of colitis. Carbohydr. Polym..

[18] Krishnan R., Stapledon C.J.M., Mostafavi H., Freitas J.R., Liu X., Mahalingam S., Zaid A. (2023). Anti-inflammatory actions of Pentosan polysulfate sodium in a mouse model of influenza virus A/PR8/34-induced pulmonary inflammation. Front. Immunol..

[19] Hu H., Sun W., Zhang L., Zhang Y., Kuang T., Qu D., Lian S., Hu S., Cheng M., Xu Y. (2024). Carboxymethylated Abrus cantoniensis polysaccharide prevents CTX-induced immunosuppression and intestinal damage by regulating intestinal flora and butyric acid content. Int. J. Biol. Macromol..

[20] Yang C., Xu Y., Gong F., Li S., Zhan H., Huang Z., Wang M., Li H., Huang H. (2025). Shengxian decoction alleviates cyclophosphamide-induced immunosuppression via improving B cell-mediated immune responses. Front. Pharmacol..

[21] Xu Q., Zhang R., Mu Y., Song Y., Hao N., Wei Y., Wang Q., Mackay C.R. (2022). Propionate Ameliorates Alcohol-Induced Liver Injury in Mice via the Gut-Liver Axis: Focus on the Improvement of Intestinal Permeability. J. Agric. Food Chem..

[22] He Y., Nong Y., Qin J., Feng L., Qin J., Wang Q., Deng L., Tang S., Zhang M., Fan X. (2024). Protective effects of oyster polypeptide on cyclophosphamide-induced immunosuppressed rats. J. Sci. Food Agric..

[23] Li Y., Zhang M., Zhang K., Niu H., Li H., Wu W. (2025). Ginsenosides modulate immunity via TLR4/MyD88/NF-κB pathway and gut microbiota. Phytomedicine.

[24] Jeong H., Koh J., Kim S., Yim J., Song S.G., Kim H., Li Y., Lee S.H., Chung Y.K., Kim H. (2025). Cell-intrinsic PD-L1 signaling drives immunosuppression by myeloid-derived suppressor cells through IL-6/Jak/Stat3 in PD-L1-high lung cancer. J. Immunother. Cancer.

[25] Chen H., Nie P., Li J., Wu Y., Yao B., Yang Y., Lash G.E., Li P. (2024). Cyclophosphamide induces ovarian granulosa cell ferroptosis via a mechanism associated with HO-1 and ROS-mediated mitochondrial dysfunction. J. Ovarian Res..

[26] Martinson M.L., Lapham J. (2024). Prevalence of Immunosuppression Among US Adults. JAMA.

[27] Feng H., Fan J., Lin L., Liu Y., Chai D., Yang J. (2019). Immunomodulatory Effects of Phosphorylated Radix Cyathulae officinalis Polysaccharides in Immunosuppressed Mice. Molecules.

[28] Sun L., Su Y., Jiao A., Wang X., Zhang B. (2023). T cells in health and disease. Signal Transduct. Target. Ther..

[29] Yang C., Xu X., Wu M., Zhao Z., Feng Y., Liang W., Xu C., Jiang T., Zhang G. (2024). Huang-Jin-Shuang-Shen Decoction promotes CD8+ T-cell-mediated anti-tumor immunity by regulating chemokine CXCL10 in gastric cancer. Phytomedicine.

[30] Tan B., Zhang J., Kang A., Zhang L., Fang D., Wu H., Han T., Qiu R., Li H., Sun D. (2025). Coptisine activates aryl hydrocarbon receptor to regulate colonic epithelial homeostasis in DSS induced ulcerative colitis and TNF-α challenged intestinal organoids. Phytomedicine.

[31] Ng C.T., Fong L.Y., Abdullah M.N.H. (2023). Interferon-gamma (IFN-γ): Reviewing its mechanisms and signaling pathways on the regulation of endothelial barrier function. Cytokine.

[32] Garbers C., Heink S., Korn T., Rose-John S. (2018). Interleukin-6: Designing specific therapeutics for a complex cytokine. Nat. Rev. Drug Discov..

[33] Bonaud A., Khamyath M., Espéli M. (2023). The cellular biology of plasma cells: Unmet challenges and opportunities. Immunol. Lett..

[34] Peng Y., Liu L., Li X., Song D., Huang D. (2025). B Cells at the Core: Immune Mechanisms and Therapeutic Potentials in Periapical Lesions. J. Endod..

[35] Raj R.S., Bonney E.A., Phillippe M. (2014). Influenza, immune system, and pregnancy. Reprod. Sci..

[36] Liu Q., Zhou Y.H., Yang Z.Q. (2016). The cytokine storm of severe influenza and development of immunomodulatory therapy. Cell Mol. Immunol..

[37] Chen J., Wang Y., Liu X., Chen M., Zang L., Wang G. (2025). ZuoJin Pill intervention in HCT116 tumor-bearing mice: Modulation of tumor growth, intestinal barrier integrity and microbial composition. Phytomedicine.

[38] Chelakkot C., Ghim J., Ryu S.H. (2018). Mechanisms regulating intestinal barrier integrity and its pathological implications. Exp. Mol. Med..

[39] Huang X., Jiang F., Chen X., Xian Y. (2024). Plant-Derived Polysaccharides Benefit Weaned Piglets by Regulating Intestinal Microbiota: A Review. J. Agric. Food Chem..

[40] Zhang W., Zhang Y., Zhao Y., Li L., Zhang Z., Hettinga K., Yang H., Deng J. (2024). A Comprehensive Review on Dietary Polysaccharides as Prebiotics, Synbiotics, and Postbiotics in Infant Formula and Their Influences on Gut Microbiota. Nutrients.

[41] Husted A.S., Trauelsen M., Rudenko O., Hjorth S.A., Schwartz T.W. (2017). GPCR-Mediated Signaling of Metabolites. Cell Metab..

[42] Vinolo M.A., Ferguson G.J., Kulkarni S., Damoulakis G., Anderson K., Bohlooly Y.M., Stephens L., Hawkins P.T., Curi R. (2011). SCFAs induce mouse neutrophil chemotaxis through the GPR43 receptor. PLoS ONE.

[43] Wu H., Singer J., Kwan T.K., Loh Y.W., Wang C., Tan J., Li Y.J., Lai S.W.C., Macia L., Alexander S.I. (2020). Gut Microbial Metabolites Induce Donor-Specific Tolerance of Kidney Allografts through Induction of T Regulatory Cells by Short-Chain Fatty Acids. J. Am. Soc. Nephrol..

[44] Ito T., Nakanishi Y., Shibata R., Sato N., Jinnohara T., Suzuki S., Suda W., Hattori M., Kimura I., Nakano T. (2023). The propionate-GPR41 axis in infancy protects from subsequent bronchial asthma onset. Gut Microbes.

[45] Trompette A., Gollwitzer E.S., Yadava K., Sichelstiel A.K., Sprenger N., Ngom-Bru C., Blanchard C., Junt T., Nicod L.P., Harris N.L. (2014). Gut microbiota metabolism of dietary fiber influences allergic airway disease and hematopoiesis. Nat. Med..

[46] Tan J.K., McKenzie C., Mariño E., Macia L., Mackay C.R. (2017). Metabolite-Sensing G Protein-Coupled Receptors-Facilitators of Diet-Related Immune Regulation. Annu. Rev. Immunol..

[47] Faas M.M., de Vos P. (2020). Mitochondrial function in immune cells in health and disease. Biochim. Biophys. Acta Mol. Basis Dis..

[48] Vardhana S.A., Hwee M.A., Berisa M., Wells D.K., Yost K.E., King B., Smith M., Herrera P.S., Chang H.Y., Satpathy A.T. (2020). Impaired mitochondrial oxidative phosphorylation limits the self-renewal of T cells exposed to persistent antigen. Nat. Immunol..

[49] Deng J., Zhang J., Chang Y., Wang S., Shi M., Miao Z. (2022). Effects of Chinese yam polysaccharides on the immune function and serum biochemical indexes of broilers. Front. Vet. Sci..

[50] Qu Q., Ma Y., Huang Y., Gao X., Xuan Z., Chen X., Tong Y., Chai S., Cao M., Dong Q. (2025). Effects of Chinese yam peels on immunity and gut microbiota in cyclophosphamide-induced chickens and optimization of extraction process. Poult. Sci..

[51] Lu J., Hao K., Song Y., Fang J., Hu B., Liu W., Hui G., Xie Y., Zhao Y. (2026). Yam-Active Protein Protects Against Cyclophosphamide-Induced Testicular Injury by Suppressing Inflammatory Responses. Molecules.

[52] Lu J., Qin H., Liang L., Fang J., Hao K., Song Y., Sun T., Hui G., Xie Y., Zhao Y. (2024). Yam protein ameliorates cyclophosphamide-induced intestinal immunosuppression by regulating gut microbiota and its metabolites. Int. J. Biol. Macromol..

[53] Yang S., Sun X., Liu D., Zhang Y., Gao X., He J., Cui M., Fu S., He D. (2024). Allantoin ameliorates dopaminergic neuronal damage in MPTP-induced Parkinson’s disease mice via regulating oxidative damage, inflammation, and gut microbiota disorder. Food Funct..

[54] Xu Y., Wang N., Tan H.Y., Li S., Zhang C., Zhang Z., Feng Y. (2020). *Panax notoginseng* saponins modulate the gut microbiota to promote thermogenesis and beige adipocyte reconstruction via leptin-mediated AMPKα/STAT3 signaling in diet-induced obesity. Theranostics.

